# Targeting programmed death ligand 2/repulsive guidance molecule b pathway: a novel strategy to subdue tumor immunotherapy resistance to anti‐programmed death 1/programmed death ligand 1

**DOI:** 10.1002/mco2.394

**Published:** 2023-10-01

**Authors:** Yuancai Xiang, Irfan Ullah, Hongming Miao

**Affiliations:** ^1^ Department of Biochemistry and Molecular Biology Southwest Medical University Luzhou China; ^2^ Department of Pathophysiology College of High Altitude Military Medicine Third Military Medical University (Army Medical University) Chongqing China; ^3^ Department of Biological Science Karakoram International University Gilgit Pakistan

1

In a recent article published in *Nature*, Park et al. revealed that good gut microbiota could improve tumor responsiveness to anti‐programmed death 1 (PD‐1) and its ligand 1 (PD‐L1) by suppressing the PD‐L2 ‐repulsive guidance molecule b (RGMb) pathway between dendritic cells (DCs) and T cells in different mouse models.^1^ They identified *Coprobacillus cateniformis* (*C. cateniformis*) as one of the good bacteria that can mediate this effect. This provides new effective intervention strategies for cancer patients who do not respond to anti‐PD‐1 or PD‐L1 therapy.

Immunotherapy has revolutionized cancer treatment in the past decade, especially the successful application of immune checkpoint inhibitors (ICIs) therapy in clinical practices that mainly enhance the host immunity via blocking co‐inhibitory molecules like PD‐1 or PD‐L1 to improve the immune‐killing effect of T cells. However, only a small part of patients benefit from them owing to resistance raised by multiple factors, including gene mutation, heterogeneity, gut microbiota, and diet.^2^ Intriguingly, the fact that gut microbiota can affect the response efficiency of ICIs provides new clues to improve cancer immunotherapy, whereas the underlying mechanism remains unknown. Recently, Park et al. provided new insight by which intervention microbiome overcome the resistance of PD‐1/PD‐L1 related immunotherapy (Figure 1).^1^

**FIGURE 1:**
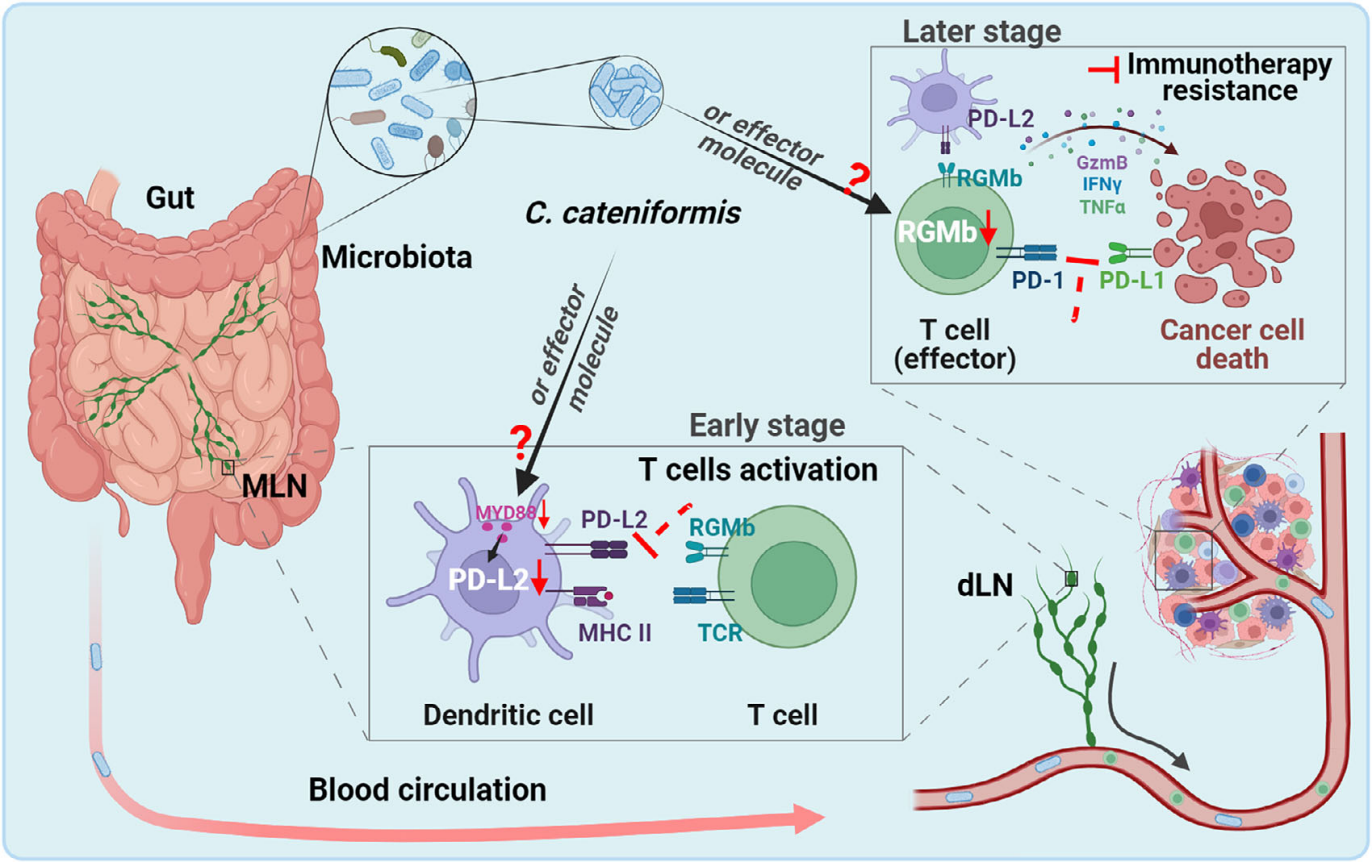
Schematic illustration of gut microbiota‐mediated anti‐tumor immunity enhancement. The anti‐tumor immunity responses to anti‐programmed death 1/programmed death ligand 1 in tumor‐beared mice were prevented without gut microbiota, which was reversed by the colonization of healthy human microbiota *C. cateniformis*. At the early stage of anti‐PD1/PD‐L1 treatment, *C. cateniformis* or its effector molecular transferred to mesenteric lymph nodes (MLNs) or tumor‐draining lymph nodes (dLNs) through blood circulation to decrease expression of PD‐L2 in dendritic cells by MYD88, thereby activating T cells by decline repulsive guidance molecule b (RGMb) signaling. Then, activated T cells were moved to tumor sites to destroy tumor cells by secretion of granzyme B (GzmB), interferon‐gamma (IFNγ), and tumor necrosis factor‐alpha (TNF‐α) when blockade PD‐1/PD‐L1. Additionally, *C. cateniformis* or its effector molecular can activate T cells in tumor sites by downregulating RGMb to clear tumor cells, especially in the presence of dendritic cells, at the later stage of anti‐PD‐1/PD‐L1 treatment. *Figure created with BioRender.com*.

In this study, to determine the effect of gut microbiota on tumor responsiveness of ICIs, Park et al. employed several groups of mice (i.e., specific pathogen‐free (SPF) mice with gut microbiome, germ‐free (GF) mice without a gut microbiome, the GF mice reconstructed by healthy human or mouse microbiota (HMB/MMB), SPF mice were depleted of gut commensals by four oral antibiotics (ABX) or reconstructed by HMB (ABX/HMB)) to implant with tumor and treated with anti‐PD‐1/PD‐L1, and found that the anti‐tumor effect was disappeared in the mice without microbiota, which was regained by HMB. By analyzing the intrinsic immunological changes of tumors at a later stage, they found that HMB could significantly increase the ratio of CD8^+^ to Treg cells and enhance T cell activity, implying HMB indeed affects T cell function in responding tumors.

To explore the underlying mechanism by which HMB boosts the anti‐tumor immunity of T cells, the immune checkpoint molecules on DCs (MHCII^+^CD11c^+^) and myeloid cells (MHCII^+^CD11b^+^) cells, within the tumor, tumor‐draining lymph nodes (dLNs), and mesenteric lymph nodes (MLNs) were examined at the early stage before the divergence of tumor size. The results showed that only PD‐L2 exhibited a significant decrease in DCs and MHCII^+^CD11b^+^ cells in dLNs and MLNs, rather than in tumors, within ABX/HMB‐treated mice, compared to those in the mice fed with ABX alone. This decrease in PD‐L2 expression was accompanied by an upregulation of PD‐L1. In contrast, the study found no significant differences in T cell effector functions in tumors between the groups treated with blocking antibodies, but there was a notable increase in the number of CD8^+^ and CD4^+^ T cells and MHCII^+^CD11b^+^ cells in the dLNs at the early stage. Of note, the researcher observed a consistent downregulation of PD‐L2 on DCs within MLNs and dLNs at the late stage. These findings suggest that HMB promotes the anti‐tumor immune response via a decrease in the abundance of PD‐L2 on DCs. It was further supported by an experiment targeting PD‐L2 to improve tumor responsiveness to ICIs in multiple mouse models. Interestingly, the mice colonized with fecal samples from melanoma patients that had either shown a complete response or had developed resistance to anti‐PD‐1 also demonstrated similar responses to ICIs. Moreover, the treatment efficiency of these resistant mice significantly increased by the combination of anti‐PD‐L2, suggesting the clinical potential of PD‐L2 in improving the ICIs response in cancer patients.

To investigate the role of microbiota in blocking PD‐1 or PD‐L1‐mediated anti‐tumor effects, authors constructed different gut commensals‐depleted mouse models with a single antibiotic that was administered orally. By using 16S rRNA sequencing of stool samples, antibiotic selection, and selective media, together with the phenotype of these groups, the authors identified two effective species of bacteria, *C. cateniformis*, and *Erysipelatoclostridium ramosum*, from HBM, whereas only *C. cateniformis* or its extracts could decline PD‐L2 expression in DCs. Moreover, in vitro assay showed that *C. cateniformis‐*induced activation of CD8^+^ T cells depended upon the downregulation of PD‐L2 in DCs. In addition, the colonization of *C. cateniformis* demonstrated similar anti‐tumor immune effects to anti‐PD‐L2 when employing combination treatment with anti‐PD‐L1 in ABX mice, and recapitulates the major advantageous immunological phenotype of HMB. More importantly, these effects were suppressed by elevating PD‐L2 expression in DCs, implying *C. cateniformis* boosts the role of anti‐PD‐L1 via decreasing DCs' PD‐L2 level.

PD‐1 is a common receptor of PD‐L1 and PD‐L2, whereas simultaneous blocking of PD‐1 and PD‐L2 obtained preferable tumor inhibition than anti‐PD‐1 alone, implying another binding partner of PD‐L2 involving these processes. Therefore, Park et al. attempted to determine the role of RGMb, another identified PD‐L2 binding protein, in promoting anti‐tumor immune effects to ICIs. Blocking PD‐L2 with different clones and depleting RGMb^+^ cells showed that only employing an antibody that specifically inhibits PD‐L2/RGMb interaction can disrupt tumor anti‐PD‐L1 resistance in germ‐free mice. Of note, these tumor therapy effects triggered by anti‐PD‐L2 were abolished when lacking CD8^+^ T cells, suggesting that CD8^+^ cells are downstream effector cells of anti‐PD‐L2. Intriguingly, the expression of RGMb was markedly increased in tumor‐infiltrating CD8^+^ T cells, but not other immune cells, in anti‐tumor immune responses resistance mice, and its anti‐tumor effect was confirmed by conditional knockout of RGMb in T cells. It is remarkable that the combination treatment of anti‐RGMb and PD‐L1 in the early stage also elevated T cells' pro‐inflammatory cytokine expression, indicating anti‐RGMb could increase T cells' anti‐tumor immunity.

In summary, the work by Park et al. revealed a novel mechanism that gut microbiota such as *C. cateniformis* improves tumor responsiveness to anti‐PD‐1/PD‐L1 through suppressing expression of PD‐L2 and RGMb in DCs and T cells, respectively. Although not all tumor mice displayed responses to monotherapy to anti‐PD‐1/PD‐L1, the PD‐L2/RGMb pathway intervention via manipulating gut microbiota exhibits anti‐tumor immunity responses, which warrants further study to validate in clinical trials. Although previous studies have demonstrated that gut microbiome can modulate local immune environment directly or indirectly through their metabolites, such as acetate, short‐chain fatty acids, and butyrate,^2^ there are some detailed mechanisms remain unknown, such as which effector molecular from *C. cateniformis* and how it regulates immune cells (DCs and T cells) function by affecting endogenous expression of PD‐L2/RGMb from gut to dLNs and tumor microenvironment (Figure 1). It's worth noting that as one of the four members of the RGM family, RGMb, originally identified as a crucial molecular in nervous system development, is ubiquitously expressed in a variety of tissues and immune cells, and binds to multiple proteins such as bone morphogenetic proteins 2 and 4, cytotoxic T‐lymphocyte associated protein 4, and neogenin to participate in multiple processes including the regulation of inflammatory factor expression, infiltration of T cells.^3^ Therefore, blocking RGMb may result in some side effects like increasing the risk of acute kidney injury.^4^ Collectively, this work provides new insight for understanding microbiome‐related immunotherapy resistance and offers innovative tactics to subdue the resistance of anti‐tumor immune therapy, which may benefit cancer patients who do not respond to anti‐PD‐1/PD‐L1 therapy.

## AUTHOR CONTRIBUTIONS

Y.X. and H.M. discussed and wrote the manuscript. I.U. and H.M. revised this manuscript. All authors have read and approved the final manuscript.

## CONFLICT OF INTEREST STATEMENT

The authors declare no conflict of interest.

## ETHICS STATEMENT

Not applicable.

## Data Availability

Not applicable.
